# Mg/Al LDH Enhances Sulfate removal and Clarification of AMD Wastewater in Precipitation Processes

**DOI:** 10.3390/ma12142334

**Published:** 2019-07-23

**Authors:** Paulina Maziarz, Jakub Matusik, Tiina Leiviskä

**Affiliations:** 1 Department of Mineralogy, Petrography and Geochemistry; Faculty of Geology, Geophysics and Environmental, Protection; AGH University of Science and Technology, al. Mickiewicza 30, 30-059 Krakow, Poland; 2 Chemical Process Engineering, University of Oulu, P.O. Box 4300, FIN-90014 Oulu, Finland

**Keywords:** acid mine drainage water, sulfate, precipitation, layered double hydroxides, calcium hydroxide

## Abstract

The sulfate removal from acid mine drainage (AMD) water (initial concentration: 5301 mg/L) was investigated by precipitation and/or adsorption using calcium hydroxide (Ca(OH)_2_) and synthetic layered double hydroxide (LDH) of the Mg/Al type. The exclusive use of LDH efficiently removed sulfates (64.2% reduction); however, alteration of its structure was observed due to low pH. The use of Ca(OH)_2_ in different doses calculated in relation to gypsum stoichiometry allowed to achieve an 86% removal of sulfates. Depending on the equilibrium pH, gypsum or ettringite were the main identified phases. The two-step removal, involving the use of Ca(OH)_2_ followed by LDH, was less efficient than the use of the Ca(OH)_2_/LDH mixture when the stoichiometric amount of Ca(OH)_2_ in relation to gypsum was applied. The application of mixture resulted in a fast pH increase, which prevented destruction of the LDH structure. Most importantly, the use of mixture significantly reduced the sludge volume and enhanced its settling velocity.

## 1. Introduction

The problem regarding acid mine drainage (AMD) water affects many countries with developed metals and the coal mining industry. The AMD wastewaters are strongly acidic with high concentration of bioavailable, toxic ions e.g., heavy metals. These substances are released to groundwater due to weathering of sulfide bearing rocks, usually mineral ores containing pyrite. Due to high levels of toxicity, connected with the presence of heavy metals and low pH, the AMD waters have a severe influence on the salinity of soils and surrounding water systems, as well as human health.

As a result of sulfides oxidation, sulfate ions are found to be the main contaminant, besides heavy metals. The problematic issue that should be taken into consideration is usually the very high concentration of sulfate in AMD water. Referring to the Polish regulations the Ministry of Investment and Economic Development, the permitted concentration of sulfates for industrial wastewaters, which could be introduced to the sewage system, is 500 mg/L [1]. Several technologies have been considered for sulfate removal, e.g., ion-exchange [2], reverse osmosis [3], electro-coagulation [4], precipitation [5], and biological treatment [6]. Among these methods, precipitation is one of the most simple and widely used. Unlike the other methods, the precipitation is very efficient regardless of the low pH of wastewater, which is a characteristic property of AMD water. The additional advantage of this method is the simultaneous removal of sulfate and heavy metals as a result of pH increase. The gypsum (CaSO_4_·2H_2_O) or ettringite (Ca_6_Al_2_(OH)_12_(SO_4_)_3_·26H_2_O) precipitation, by the addition of solid calcium hydroxide (Ca(OH)_2_), is a well-known method of sulfate removal from wastewaters. However, the formed precipitate usually requires much time to settle without the addition of coagulants. In addition, the solid precipitates have to be stored in a restricted area. The important issue that has to be considered in the case of sulfate precipitation is the limited solubility of Ca(OH)_2_, as well as the relatively high solubility of the formed gypsum (1500–2000 mg/L).

The layered double hydroxides (LHDs), are well-known as very efficient adsorbents in the removal of anionic pollutants [7,8,9]. Their general chemical formula can be expressed as [M_1−x_^2+^M_x_^3+^(OH)_2_]^x+^(A^n−^)_x/n_·mH_2_O, where M^2+^ and M^3+^ are divalent and trivalent metal cations, respectively, A^n−^ is an anions, and x is a molar ratio of M^3+^ to total metal [10]. The LDH structure is made of positively charged brucite-like layers and charge-balancing hydrated anions, located in the interlayer space. However, the LDH materials are easily dissolved at low pH. This significantly hinders the possibility of LDH application in AMD water treatment.

Therefore, in this research, the novel approach of sulfate removal from AMD water was to investigate precipitation and adsorption, using Ca(OH)_2_ and LDH in a two-step process (precipitation followed by adsorption) and simultaneous treatment. The application of Ca(OH)_2_ was aimed at the precipitation of sulfates as well as increasing the pH, which enables the use of LDH in the enhancement of sulfate removal. The studies were carried out to evaluate the optimal dosages of the two components in a mixture for the most efficient sulfate removal from real AMD water. The possible synergistic effects behind using Ca(OH)_2_ and LDH were investigated. The kinetic results allowed us to estimate the time needed to reach the equilibrium concentration of sulfates. The precipitated solid phases were characterized using X-ray diffraction (XRD), Fourier transformed infrared spectroscopy (FTIR), and scanning electron microscopy (SEM).

## 2. Materials and Methods 

### 2.1. Materials 

All of the reagents used in the experiments were of analytical grade, including the calcium hydroxide (Ca(OH)_2_), magnesium chloride (MgCl_2_·6H_2_O), aluminum chloride (AlCl_3_·6H_2_O), and sodium hydroxide (NaOH). The AMD water was collected from Podwiśniówka quartzite quarry. The quarry is located in the western part of the Świętokrzyskie Mountains, near Kielce city, in Poland. The initial concentration (C_in_) of sulfate in AMD water is equal to 5301.6 mg/L and the initial pH (pH_in_) is 2.20.

### 2.2. LDH Synthesis

The LDH synthesis was carried out using a chemical precipitation method. As the sources of Mg(II) and Al(III), MgCl_2_۰6H_2_O and AlCl_3_۰6H_2_O were used, respectively. The molar ratio of Mg:Al was 2:1. Firstly, the solution of Mg and Al was prepared. In the next step, the pH of the solution was raised and controlled in the range of 9–10 by dropwise addition of 4 mol/L NaOH. Afterwards, the resulting white suspension was aged for 24 h, washed with deionized water, and dried at 60 °C for 24 h.

### 2.3. Sulfate Removal

In all experiments, the samples were shaken for 24 h in room temperature (22 °C) and then centrifuged at 4500 rpm for 10 min. The sulfate concentration was analyzed using an inductively coupled plasma-optical emission spectrometer (ICP-OES, Thermo Scientific, iCAP 6500 Duo, measured as sulfur).

#### 2.3.1. The Sulfate Removal by LDH, Ca(OH)_2,_ and a Two-Step Experiment

In the first experiment, the effect of the LDH dosage (2–20 g/L) on the sulfate removal was studied. In the second experiment, Ca(OH)_2_ was used to remove sulfate from AMD water by precipitation method. The stoichiometric amount (SA) of Ca(OH)_2_ was calculated, taking into account the sulfate content and assuming that gypsum will precipitate as a sole phase. Additionally, the twice (2SA) and three times (3SA) the stoichiometric amounts of Ca(OH)_2_ were used. The residual sulfate was then removed using LDH (2–20 g/L).

#### 2.3.2. The Sulfate Removal by a Mixture of Ca(OH)_2_ and LDH.

In the third experiment, simultaneous removal of sulfate by precipitation with Ca(OH)_2_ and adsorption by LDH was carried out. Before application of the solid phases for sulfate removal from AMD water, the appropriate amounts of Ca(OH)_2_ and LDH were physically mixed to ensure homogeneity of the solid phase. The SA and 2SA dosages of Ca(OH)_2_ and 5 g/L of LDH were chosen on the basis of the results obtained from the Ca(OH)_2_ and LDH dosage experiment. 

#### 2.3.3. The Kinetic Experiment.

The kinetics of the sulfate removal was investigated for untreated AMD. The experiments were carried out for LDH (5 g/L), SA, and 2SA, as well as the physical mixtures of SA+LDH and 2SA+LDH. The samples were collected after certain time intervals (0–24 h) and afterwards immediately filtered through a 0.2 µm membrane. The sulfate concentration was analyzed using the turbidimetric method [11].

### 2.4. Analysis of Solid Sedimentation Rate 

The sedimentation experiments were carried out using six-jar (1000 mL) paddle stirrer equipment from Kemira Kemwater (Flocculator, 2000). A 700 mL of AMD was used in the sedimentation experiment. The experiments were carried out for SA and 2SA, as well as mixtures of SA+LDH and 2SA+LDH. The following mixing and settling programme was used: 300 rpm rapid for 30 s, 80 rpm slow mixing for 180 min, and settling for 30 min. Afterwards, the supernatant was collected at a depth of ~4 cm below the surface. The turbidity of the suspension was analyzed immediately (Hach Ratio XR, Hach Company). Additionally, the amount of formed sludge volume was studied for SA and the mixture of SA+LDH. The samples were mixed for 1 h and then allowed to settle in a 50 mL polyethylene test tube.

### 2.5. Characterization Methods

The resulted solid precipitates were characterized by powder X-ray diffraction (XRD) using a Rigaku Miniflex 600 diffractometer with a Cu-Kα radiation source (λ = 0.15432 nm). The XRD patterns were recorded in the range 2–70°2θ with a scan step of 0.05°2θ. The FTIR spectra were obtained in the region 400–4000 cm^−1^ with a Thermo Scientific Nicolet 6700 spectrometer. The pellets were prepared with a spectroscopic grade KBr and measured using a transmission mode (64 scans at 4 cm^−1^ resolution). The morphology of the precipitates was observed with a FEI Quanta 200 FEG scanning electron microscope.

## 3. Results and Discussion.

### 3.1. The Sulfate Removal by the LDH: Dosage Experiment

**Figure 1:**
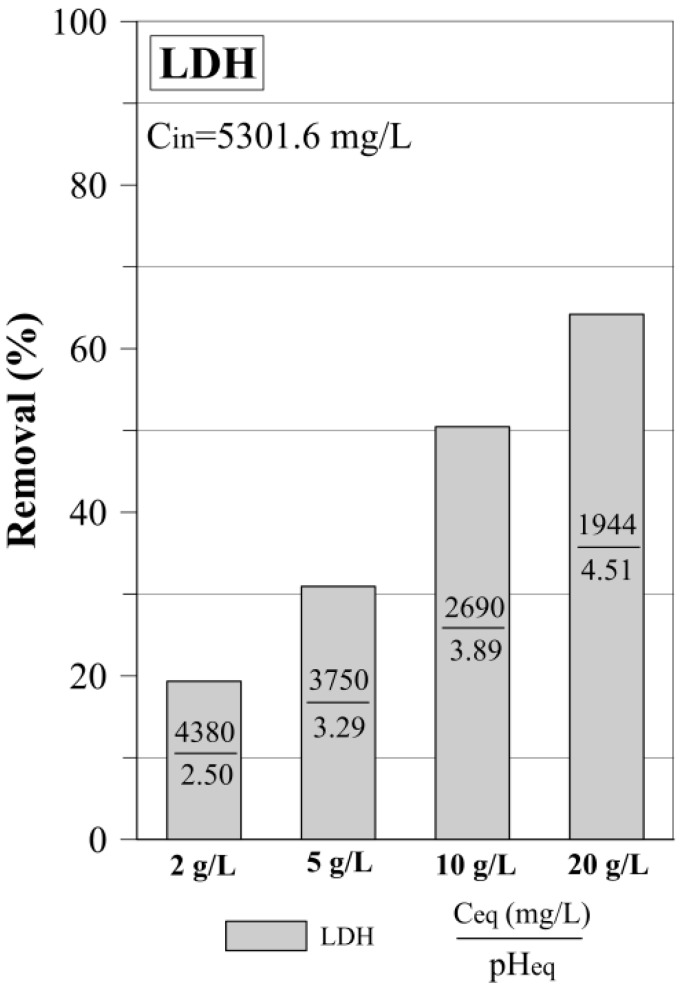
Sulfate removal by layered double hydroxide (LDH) in the function of dosage.

As the preliminary experiment, the dosage experiment using only LDH was carried out. It revealed that the 2 g/L dosage of the LDH was able to remove only 19.3% of sulfate from the AMD water, which resulted in C_eq_ of sulfates equal to 4380 mg/L (Figure 1). With the increase of dosage, the efficiency of sulfate removal increased gradually up to 64.2% for 20 g/L. In this case, the C_eq_ of sulfates was lowered to 1944 mg/L.

**Figure 2:**
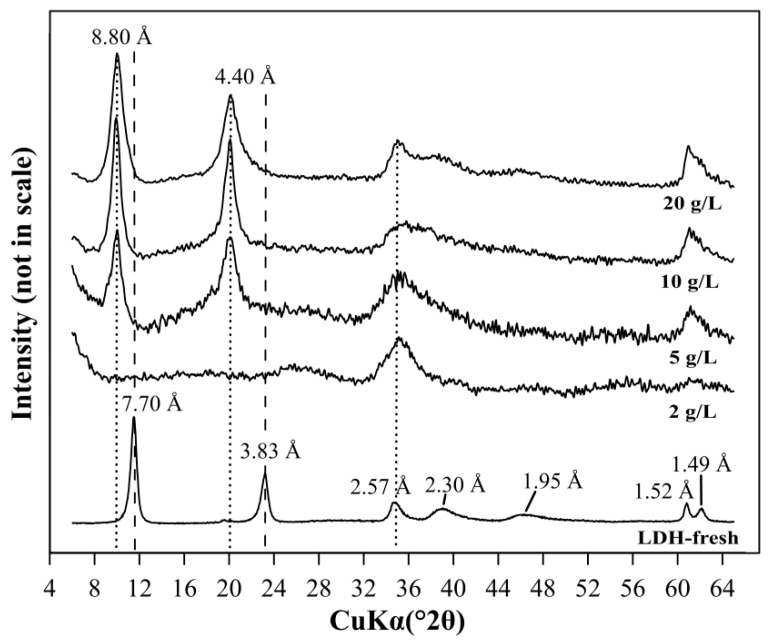
The XRD patterns of LDH before acid mine drainage (AMD) treatment (LDH-fresh) and LDH after AMD treatment in the function of dosage.

The XRD pattern of LDH solids after the AMD water treatment at a dosage of 2 g/L showed the disappearance of diagnostic peaks of LDH (7.70 Å and 3.83 Å) (Figure 2). This revealed partial dissolution of the LDH phase as a result of low pHeq 2.50. In the case of 5, 10, and 20 g/L dosages, where the pHeq was higher (3.29–4.51), the diagnostic peaks were not significantly altered. However, the XRD patterns showed the shift of LDH peaks from 7.70 Å and 3.83 Å, to 8.8 Å and 4.40 Å, respectively. These shifts are consistent with incorporation of sulfate anions into the interlayer space of LDH [12]. 

**Figure 3:**
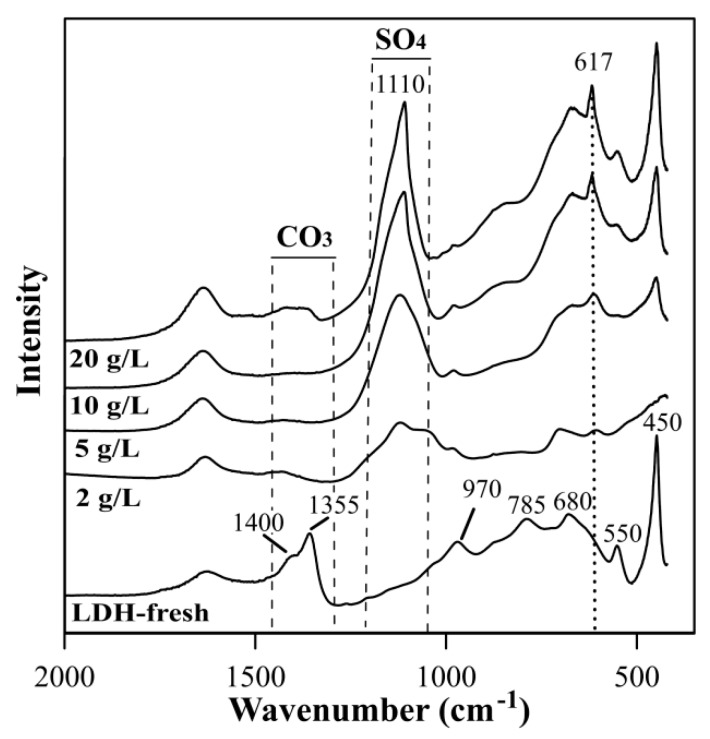
The FTIR spectra of LDH before AMD treatment (LDH-fresh) and LDH after AMD treatment in the function of dosage.

The FTIR of fresh LDH showed bands at 1480–1300 cm^−1^, which revealed the presence of carbonates in the interlayer space (Figure 3). After the AMD treatment, the appearance of band from sulfate anions was observed in the 1250–1040 cm^−1^ region with the maximum at 1110 cm^−1^. The intensity of this band increased with the LDH dosage, as a result of higher sulfate removal efficiency. The same trend was observed for a band at 617 cm^−1^, which can also be from a sulfate. Moreover, the disappearance of the band attributed to the carbonates was observed, which confirms the anion exchange as a mechanism of the sulfate removal. The FTIR spectra also showed the alteration of metal-oxygen bonds in the brucite-like lattice (900–400 cm^−1^). This was the most evident, in case of dosage of 2 g/L, which is in agreement with XRD results.

**Figure 4:**
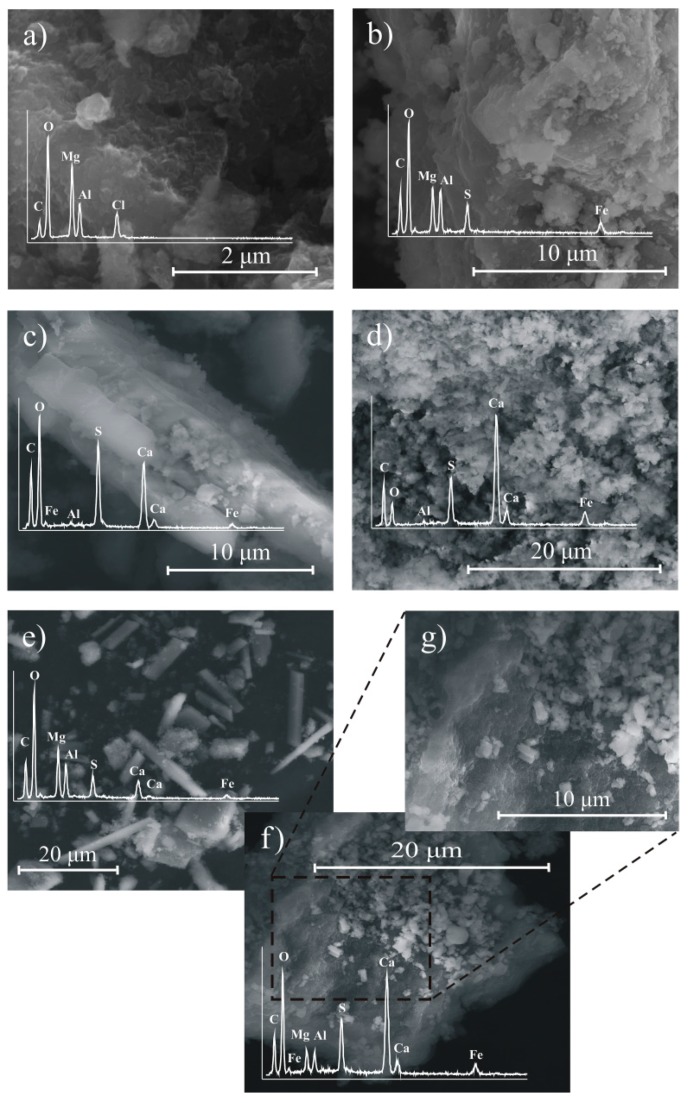
The SEM images of: (**a**) Fresh LDH, (**b**) LDH after AMD treatment (20 g/L), and solid phase resulted from AMD treatment by (**c**) the stoichiometric amount (SA), (**d**) 2SA, (**e**) SA+LDH, (**f**,**g**) twice the stoichiometric amount (2SA)+LDH.

The SEM image showed that the fresh LDH sample was composed of agglomerated small plate-like particles, which is typical for the LDH (Figure 4a). After the AMD treatment, the formation of fissures and cracks, as well as aggregation, of LDH particles was noticed, which is a result of partial LDH dissolution (Figure 4b). The EDS analysis also revealed the presence of S and Fe. This showed that, besides the sulfate, the LDH was also able to remove Fe from AMD water.

### 3.2. The Two-Step Sulfate Removal by Ca(OH)_2_ and LDH

#### 3.2.1. The Sulfate Removal by Precipitation, Using Ca(OH)_2_: Dosage Effect

**Figure 5:**
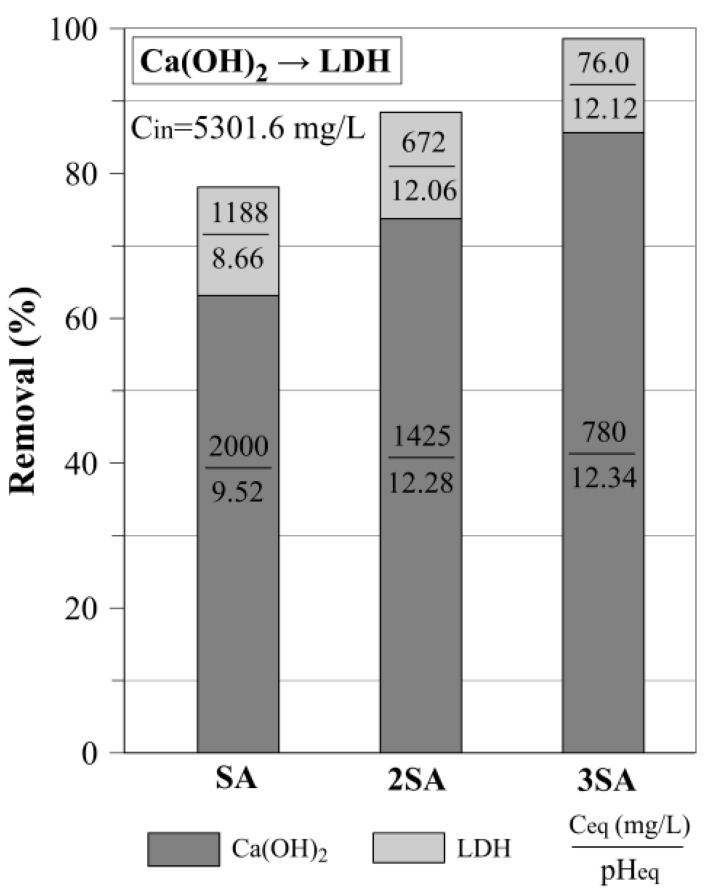
Sulfate removal in two-step treatment method (precipitation with calcium hydroxide (Ca(OH)_2_) and subsequent removal of residual sulfate by LDH (dosage 5g /L) in the function of Ca(OH)_2_ dosage.

The dosage experiment with Ca(OH)_2_ showed that SA is able to remove 63.2% of sulfate (Figure 5). With the increase of Ca(OH)_2_ dosage, the sulfate removal increased up to 85.6% for 3SA, which corresponds to a sulfate concentration decrease to 780 mg/L.

**Figure 6:**
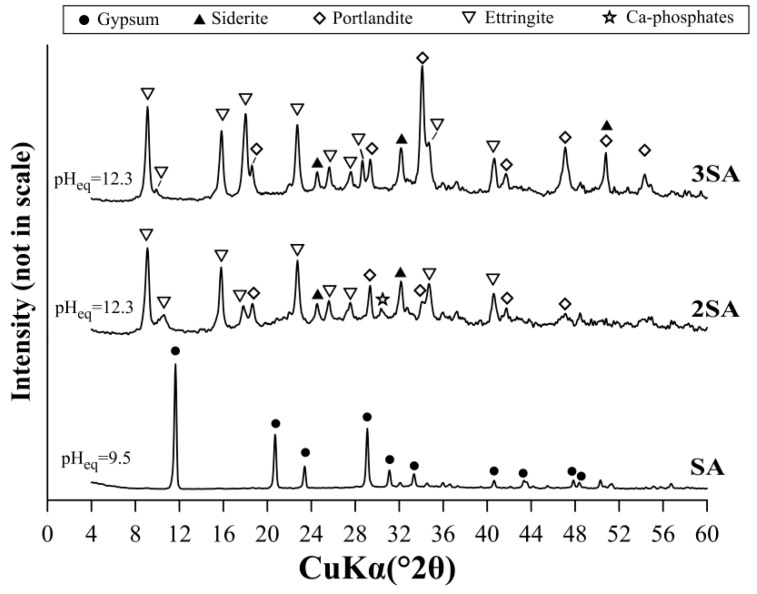
The XRD patterns of solid phases precipitated as a result of AMD treatment by SA, 2SA, and 3SA.

The mechanism of sulfate removal was precipitation since the diffraction XRD pattern revealed only the presence of peaks attributed to the gypsum (CaSO_4_·2H_2_O) in the solid phase (Figure 6). In the case of 2SA and 3SA, the peaks of the gypsum were not found. Instead, the peaks of ettringite (Ca_6_Al_2_(SO_4_)_3_(OH)_12_·26H_2_O) were observed. The differences in the type of precipitated phases resulted from the pH_eq_, which was found to be ~9.50 for SA and ~12.3 for both 2SA and 3SA. The peaks attributed to siderite (FeCO_3_), portlandite (Ca(OH)_2_), and Ca-phosphates were also found with 2SA and 3SA. The presence of siderite and Ca-phosphates was due to high content of Fe and P in the AMD water, respectively (Table S1), while the peaks of Ca(OH)_2_ resulted from the high dosage used and incomplete dissolution of Ca(OH)_2_.

**Figure 7:**
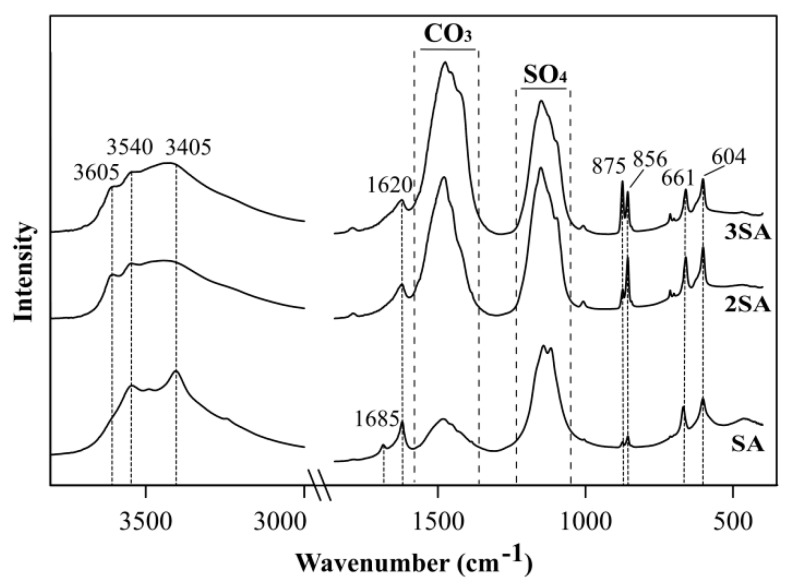
The FTIR spectra of solid phases precipitated as a result of AMD treatment by SA, 2SA, and 3SA.

The FTIR spectra of the SA showed characteristic bands of gypsum (Figure 7). Major bands corresponded to stretching vibrations of structural water molecules, characteristic for gypsum—3540 cm^−1^ and 3405 cm^−1^ [13]. Additionally, the spectra of 2SA and 3SA also showed a band at 3605 cm^−1^, which can be from a stretching vibration of the Al-OH or Ca-OH groups [13]. The O-H bending vibrations characteristic of gypsum were identified at 1685 cm^−1^ and 1620 cm^−1^. These two peaks indicate the presence of two types of water in the sample. The first band is due to water linked with the sulfate group through hydrogen bonding, whereas the second type is attributed to the direct link of water with the calcium ions (anion water) [14]. The bands of the sulfate group can be assigned between 1270–1030 cm^−1^. The bands representing the stretching and bending modes of the sulfate group can also be found at 661 cm^−1^ and 604 cm^−1^ [15]. The vibrational bands at 1590–1315 cm^−1^ and a narrow band around 875 cm^−1^ revealed the presence of CO_3_^2−^ because of the absorbed CO_2_ from the atmosphere and subsequent precipitation of carbonates. The presence of the band at 856 cm^−1^ was due to the bending vibrations of Al-O-H groups.

The SEM image of SA showed particles of prismatic structure, typical of gypsum (Figure 4c). This was also consistent with the EDS analysis. The admixture of other phases of plate-like structure was also observed. The images of 2SA and 3SA were similar. In this case, the morphology of the solid was significantly different compared with the SA; however, the needle-shape structure, typical of ettringite, was not so evident (Figure 4d). This was because of fast crystallization, which hampered formation of well-developed crystals. Moreover, the crystal growth was probably disturbed by other elements present in the AMD water in high concentration.

#### 3.2.2. The Use of LDH for Sulfate Removal After Precipitation with Ca(OH)_2_

**Figure 8:**
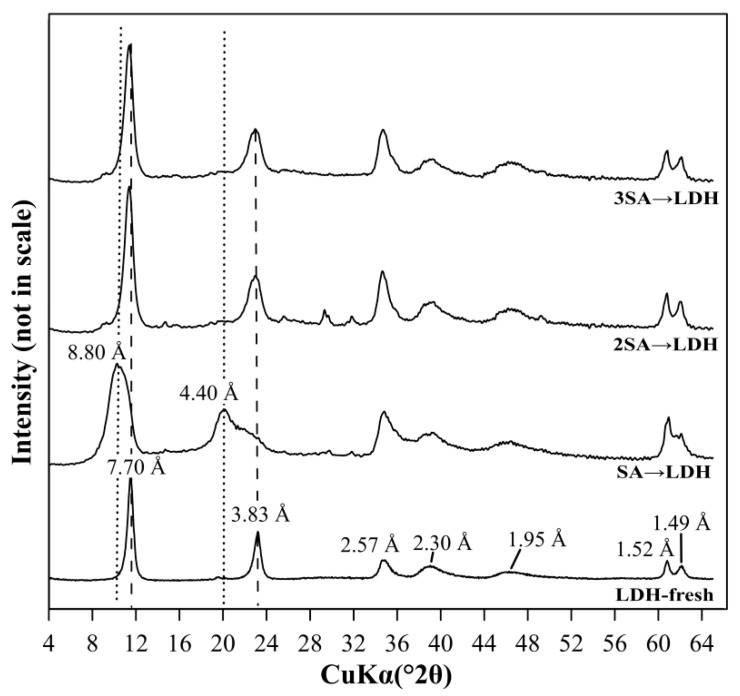
The XRD patterns of LDH, before (LDH-fresh) and after AMD treatment in a two-step treatment process.

A dosage of 5 g/L was chosen based on the LDH experiments (Section 3.1) to remove residual sulfate from AMD water after precipitation with Ca(OH)_2_. The results showed that the highest sulfate removal was 98% (C_eq_= 76 mg/L) and it was reached for 3SA and LDH (Figure 5). However, it is worth noticing that as much as 78% of the sulfate was already removed using SA and LDH. In this case, the C_eq_ of sulfates was equal to 1188 mg/L. The XRD pattern of LDH after this two-step AMD treatment showed that the layered structure of LDH was not disturbed (Figure 8). This was due to high pH_eq_ of AMD water after the application of Ca(OH)_2_ in the previous step. The broadening of LDH diagnostic peaks was observed as a consequence of sulfate incorporation into the interlayer space (8.8 Å and 4.40 Å).

### 3.3. The Sulfate Removal Using a Mixture of Ca(OH)_2_ and LDH

**Figure 9:**
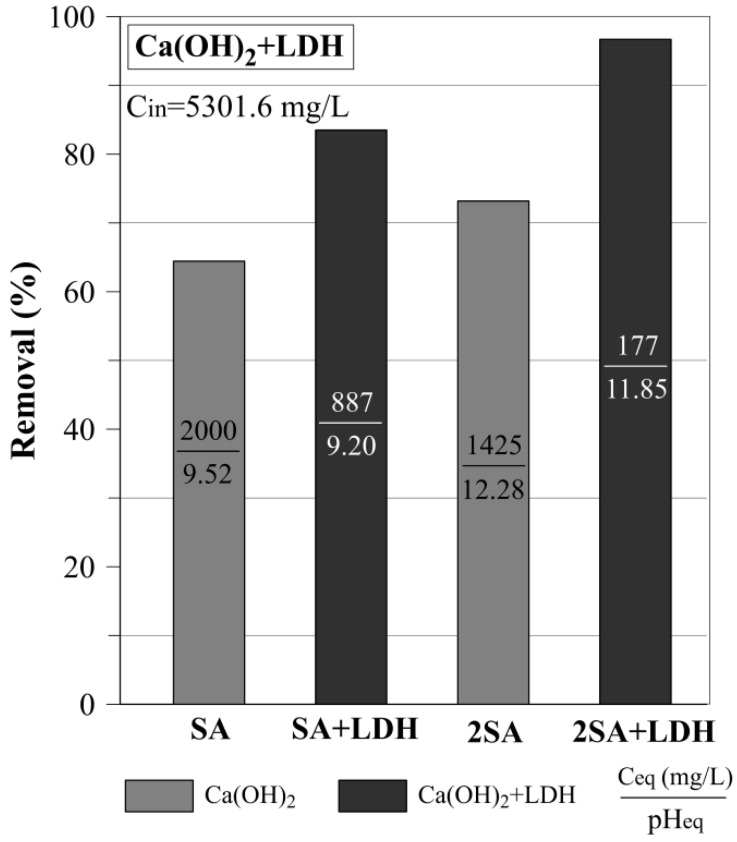
Sulfate removal using a physical mixture of Ca(OH)_2_ and LDH (SA+LDH, 2SA+LDH).

In the next step of the experiment, the sulfate removal using a physical mixture of Ca(OH)_2_ and LDH was investigated. The results showed that the mixture of 2SA+LDH (5 g/L) removed 99.5% sulfates with the equilibrium concentration of 177 mg/L (Figure 9). While the sulfate removal efficiency using a mixture of SA and LDH was equal to 83.5%. In this case, the sulfates equilibrium concentration was equal to 887 mg/L. It is also worth noticing that the efficiency of the sulfate removal using a mixture of SA+LDH was higher in comparison to the 2SA. However, the C_eq_ in the case of SA+LDH was higher than the limit of 500 mg/L, recommended by Polish law, and it should be emphasized that the C_in_ of the sulfate was very high (5301.6 mg/L) in the studied AMD water.

**Figure 10:**
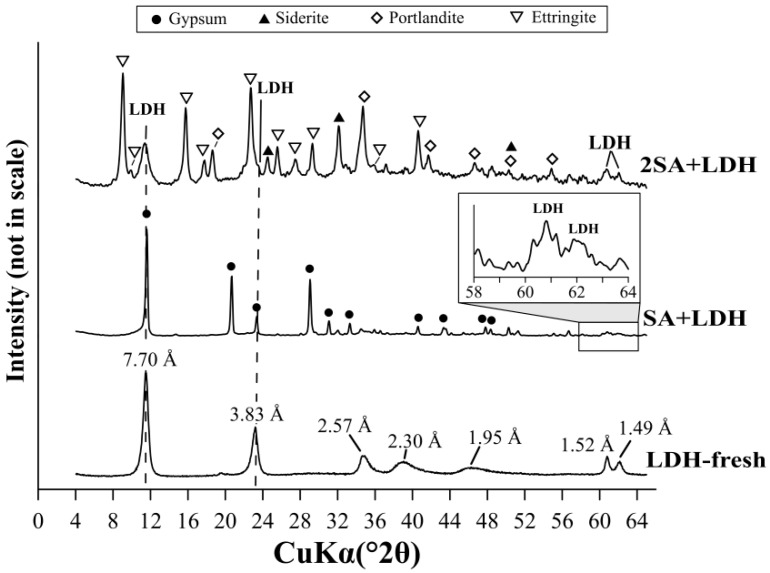
The XRD patterns of solid phases formed as a result of AMD treatment by mixtures of SA+LDH, 2SA+LDH, and LDH, before AMD treatment, as a reference sample.

The XRD of solid samples after AMD treatment by SA+LDH revealed peaks, characteristic for gypsum (Figure 10). The diagnostic peaks of LDH were not evident due to the coincidence with gypsum peaks of high intensity. The evidence for the presence of LDH in the solid samples can be seen in the XRD pattern in the region of 60–63°2θ. The characteristic plate-like particles of LDH was also observed on the SEM image (Figure 4e). Additionally, the prismatic particles were recognized as gypsum. This revealed that LDH retained its morphology after AMD treatment. The XRD pattern of 2SA+LDH showed peaks characteristic for ettringite, as well as siderite and portlandite, similar to the solid residue, when only 2SA was used. However, in the case of 2SA+LDH, the peaks of LDH were observed at 7.70 Å, 3.83 Å, 1.52 Å, and 1.49 Å. The SEM images showed the needle-shape structure precipitated on the LDH surface, recognized as ettringite.

### 3.4. Kinetics Studies of Sulfate Removal

**Figure 11:**
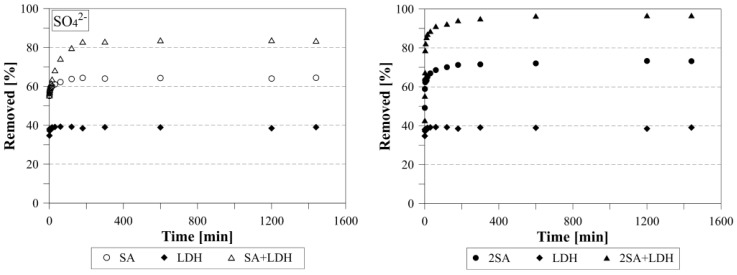
The kinetics of sulfate removal.

The kinetic studies showed that, in the case of LDH the equilibrium concentration of the sulfate was reached after 1 min (Figure 11). However, the removal efficiency was only ~39%. The kinetic studies were also carried out for SA and 2SA. The results revealed, slower kinetics, with the equilibrium achieved after 2 h and 3 h, respectively. In the case of mixtures, the equilibration time was similar, but the adsorption efficiency for the SA+LDH and 2SA+LDH was much higher and equal to 83.5% and 96.7%, respectively.

### 3.5. Sedimentation and Sludge Volume Reduction

**Figure 12:**
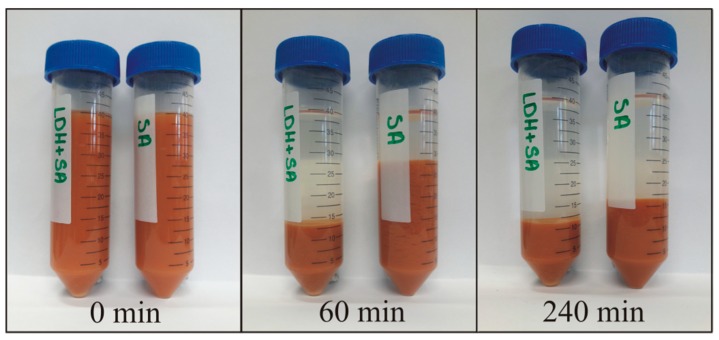
The sedimentation behavior of solid residues versus time for SA+LDH (left tube) and SA (right tube).

The settling experiments showed that after application of mixtures, the treated AMD water solutions had a greater clarity. These revealed better settlement properties of the formed sludge and synergistic effects behind the application of Ca(OH)_2_ and LDH as a mixture. According to WHO standards, the turbidity of drinking water should be below 5 NTU and preferably below 1 NTU (WHO, 2011). The lowest turbidity measured for the SA+LDH was equal to 1.1 NTU (Table 1). The application of mixtures additionally allows us to reduce the volume of formed sludge (Figure 12). It should be emphasized that the experiments revealed a more effective removal of sulfate when Ca(OH)_2_ and LDH were used in the mixture in comparison to the results obtained where only Ca(OH)_2_ was used.

## 4. Conclusions

The study revealed advantages of Ca(OH)_2_ and LDH application as a mixture in sulfate removal from AMD water. The first experiment revealed an increase of sulfate removal with the increase of LDH dosage. The highest removal was equal to 64.2% for a dosage of 20 g/L (Table 2). The shift of LDH diagnostic XRD peaks was observed as a result of the incorporation of sulfates into the interlayer space. Moreover, according to XRD and FTIR, the LDH structure was altered due to low pH_in_, as well as low equilibrium pH of the AMD water. When testing the Ca(OH)_2_, the increase of sulfate removal was also observed with the increase of the Ca(OH)_2_ dosage. However, due to low solubility, the undissolved Ca(OH)_2_ was also found in the final sludge. The treatment with Ca(OH)_2_ and LDH as a physical mixture revealed higher efficiency of sulfate removal in comparison to the experiment where two-step treatment (first Ca(OH)_2_ and then LDH) was used. The fast increase of pH as a result of Ca(OH)_2_ application prevented the destruction of the LDH structure. This resulted in effective removal of sulfate by simultaneous precipitation and adsorption. The equilibrium concentrations of sulfates were equal to 877 mg/L and 177 mg/L for the SA+LDH and 2SA+LDH mixtures, respectively. Moreover, it turned out that the applied methodology leads to a significant removal of other metals (Table S1). In particular, the initial high iron content (1116 mg/L) was found below detection. The application of mixtures also resulted in a significant reduction of sludge volume in comparison to the experiment when only Ca(OH)_2_ was used. Additionally, the settling was significantly enhanced, which could be observed by lower turbidity in the treated AMD water.

## Figures and Tables

**Table 1:** Turbidity measurements.

Sample	Turbidity after 30 min of Settling [NTU]
2SA	66.3
SA	10.8
2SA+LDH	6.2
SA+LDH	1.1

**Table 2:** The summary of results obtained for the LDH dosage experiment, two step experiment (Ca(OH)_2_→LDH), and Ca(OH)_2_+LDH mixture.

Sulfate Removal (%)			
LDH Dosage Experiment			
2 g/L	**19.3**		
5 g/L	**30.9**		
10 g/L	**50.4**		
20 g/L	**64.2**		
Two step experiment (Ca(OH)_2_→LDH)			
	Ca(OH)_2_	LDH	Σ
SA→LDH	63.1	15.0	**78.1**
2SA→LDH	73.7	14.7	**88.4**
3SA→LDH	85.6	13.0	**98.6**
Ca(OH)_2_+LDH mixture			
SA+LDH	**83.5**		
2SA+LDH	**96.7**		

* The bolded results show total sulfate removal.

## Supplementary Materials

The following are available online at https://www.mdpi.com/1996-1944/12/14/2334/s1, Table S1: The concentration of elements in the AMD water before and after treatment with SA+LDH measured with inductively coupled plasma-optical emission spectrometer (ICP-OES)/mass spectrometry (ICP-MS).
