# Supplementary material for: Mg/Al LDH Enhances Sulfate removal and Clarification of AMD Wastewater in Precipitation Processes
Source: Materials (Basel). 2019 Jul 23;12(14):2334. doi: 10.3390/ma12142334 (PMC6679161; doi:10.3390/ma12142334)
Supplement: Supplementary file 1 [file materials-12-02334-s001.pdf]

materials

Supplementary data

Mg/Al LDH Enhances Sulfate removal and Clarification of AMD Wastewater in Precipitation Processes

Paulina Maziarz [1,*], Jakub Matusik [1] and Tiina Leiviskä [2]

1 AGH University of Science and Technology; Faculty of Geology, Geophysics and Environmental, Protection; Department of Mineralogy, Petrography and Geochemistry, al. Mickiewicza 30, 30-059 Krakow, Poland, jmatusik@agh.edu.pl
2 University of Oulu, Chemical Process Engineering, P.O. Box 4300, FIN-90014 University of Oulu, Oulu, Finland, tiina.leiviska@oulu.fi
* Correspondence: pmaziarz@agh.edu.pl

Table S1. The concentration of elements in the AMD water before and after treatment with SA+LDH measured with Inductively Coupled Plasma - Optical Emission Spectrometer (ICP-OES)/ Mass Spectrometry (ICP-MS).

	AMD water		
	**Before treatment (mg/L)**	**After treatment (mg/L)**	**Detection limit (mg/L)**
Al*	75.66	2.07	0.01
As**	19.662	0.021	0.001
Ca**	216.9	591.4	0.05
Cd**	0.004	<0.0003	0.0003
Co*	1.495	0.0009	0.0002
Cr**	0.856	0.005	0.005
Cu**	5.069	0.007	0.001
Fe*	1116	<0.01	0.01
Mg*	23.18	0.845	0.1
Mn*	1.81	<0.005	0.005
Na*	6.19	12.25	0.1
Ni**	0.959	0.002	0.001
P**	0.930	<0.0061	0.0061
Sb**	0.325	<0.0002	0.0002
Si*	69.75	<0.1	0.1
Tl**	0.004	0.0013	0.0001
V**	0.205	<0.001	0.001
Zn*	0.39	0.0750	0.01
Zr**	0.011	<0.002	0.002

*ICP-OES ; **ICP-MS
